# USP7 and TDP-43: Pleiotropic Regulation of Cryptochrome Protein Stability Paces the Oscillation of the Mammalian Circadian Clock

**DOI:** 10.1371/journal.pone.0154263

**Published:** 2016-04-28

**Authors:** Arisa Hirano, Tomoki Nakagawa, Hikari Yoshitane, Masaaki Oyama, Hiroko Kozuka-Hata, Darin Lanjakornsiripan, Yoshitaka Fukada

**Affiliations:** 1 Department of Biological Sciences, School of Science, The University of Tokyo, 7-3-1 Hongo, Bunkyo-ku, Tokyo, 113–0033, Japan; 2 Medical Proteomics Laboratory, Institute of Medical Science, The University of Tokyo, 4-6-1 Shirokanedai, Minato-ku, Tokyo, 108–8639, Japan; University of Lübeck, GERMANY

## Abstract

Mammalian Cryptochromes, CRY1 and CRY2, function as principal regulators of a transcription-translation-based negative feedback loop underlying the mammalian circadian clockwork. An F-box protein, FBXL3, promotes ubiquitination and degradation of CRYs, while FBXL21, the closest paralog of FBXL3, ubiquitinates CRYs but leads to stabilization of CRYs. *Fbxl3* knockout extremely lengthened the circadian period, and deletion of *Fbxl21* gene in *Fbxl3*-deficient mice partially rescued the period-lengthening phenotype, suggesting a key role of CRY protein stability for maintenance of the circadian periodicity. Here, we employed a proteomics strategy to explore regulators for the protein stability of CRYs. We found that ubiquitin-specific protease 7 (USP7 also known as HAUSP) associates with CRY1 and CRY2 and stabilizes CRYs through deubiquitination. Treatment with USP7-specific inhibitor or *Usp7* knockdown shortened the circadian period of the cellular rhythm. We identified another CRYs-interacting protein, TAR DNA binding protein 43 (TDP-43), an RNA-binding protein. TDP-43 stabilized CRY1 and CRY2, and its knockdown also shortened the circadian period in cultured cells. The present study identified USP7 and TDP-43 as the regulators of CRY1 and CRY2, underscoring the significance of the stability control process of CRY proteins for period determination in the mammalian circadian clockwork.

## Introduction

Circadian rhythms are observed in broadly across organisms from bacteria to mammals. These rhythms are generated by an internal time-measuring system, the circadian clock, operating at the cellular level [1]. Mammalian circadian clockwork is composed of a series of clock genes and protein products forming a transcriptional-translational negative feedback loop [2]. A heterodimer of CLOCK and BMAL1 binds to E-box *cis*-elements and activates transcription of their neighboring genes [3–5]. Among those, *Period* (*Per1-3*) and *Cryptochrome* (*Cry1 and Cry2*) encode transcriptional repressors PERs and CRYs that form complexes repressing their own transcription activated by CLOCK-BMAL1 through E-box [6]. The protein levels of CRYs and PERs are strictly regulated by multiple processes, particularly posttranslational modifications. CRY1 and CRY2 have stronger repressor activities as compared to their binding partners, PER proteins [6]. Hence, the protein modifications such as phosphorylation [7–10] and ubiquitination [11] of CRY1 and CRY2 play critical roles in the circadian clockwork. For example, an F-box-type ubiquitin E3 ligase, FBXL3, ubiquitinates CRY1 and CRY2, leading to proteasomal degradation [12–14]. *Fbxl3* mutant or knockout mice [12,14–16] showed extremely long periods of the circadian rhythms at the behavioral and cellular levels. FBXL21, the closest paralog of FBXL3, also ubiquitinates and stabilizes CRY proteins [15,17]. FBXL21 functionally competes with FBXL3, and deletion of *Fbxl21* gene attenuated the period-lengthening effect of *Fbxl3* knockout in the mouse behavioral rhythms [15]. Importantly, some of the double knockout mice showed arrhythmic behaviors in constant darkness, indicating that regulation of CRY stabilities by the two ubiquitinating enzymes is crucial for the stable and robust circadian oscillation [15]. However, it is poorly understood how FBXL21 antagonizes FBXL3, and we consider that a more global network of protein-protein interactions underlies the regulation of CRY stability.

The present study aimed at identifying regulators of the protein lifetimes of CRY proteins. For this purpose, we performed a shotgun proteomics analysis of the CRY interactome. In a screen of proteins regulating CRYs stabilities, we found that ubiquitin-specific protease 7 (USP7) and TAR DNA binding protein 43 (TDP-43) stabilize CRY proteins. USP7 is a USP family deubiquitinating enzyme originally identified as herpesvirus-associated ubiquitin-specific protease (HAUSP) [18]. A research group very recently reported that USP7 regulates cellular response to DNA damage *via* CRY1 deubiquitination and stabilization [19]. Here, we found that USP7 stabilizes both CRY1 and CRY2 proteins by deubiquitination, regulating the circadian oscillation. Specifically, the inhibition of USP7 shortened the period length of the circadian clock in cultured cells. Also we found that TDP-43 associates with both CRY1 and CRY2, although TDP-43 is well known as an RNA-binding protein regulating mRNA metabolism [20,21]. Similar to USP7, TDP-43 stabilizes CRY proteins and its knockdown shortened the period length of the cellular clock. Interestingly, the stabilization of CRYs by USP7 was not affected by *Fbxl3* knockdown, while the stabilization by TDP-43 was abrogated by *Fbxl3* knockdown, suggesting that TDP-43 interferes with FBXL3 function. These results highlight a global protein network for regulation of the lifetimes of CRY1 and CRY2, and this regulatory network plays a key role for the period determination of the circadian clock.

## Results

### USP7 deubiquitinates CRY proteins

**Fig 1:**
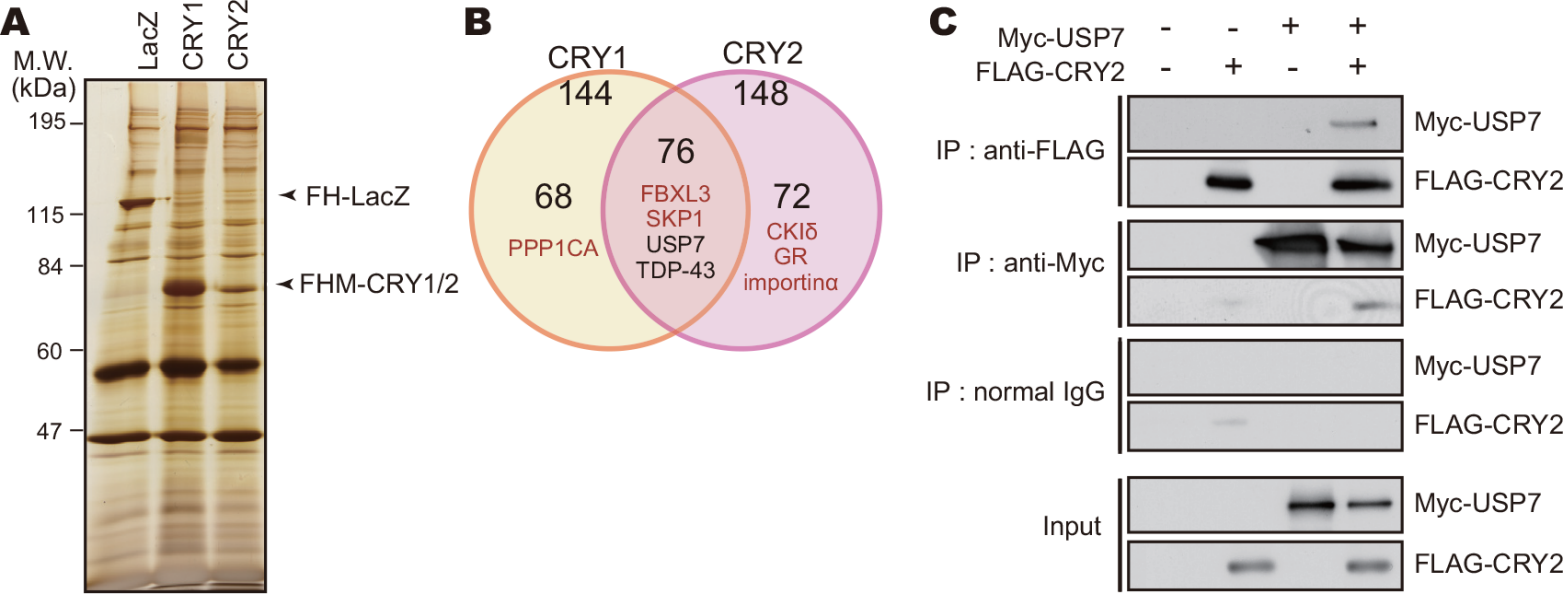
USP7 interacts with CRY proteins. **A.** Silver staining image of proteins co-purified with FLAG-His-Myc-CRY1 (FHM-CRY1) or FHM-CRY2. NIH3T3 cells expressing FHM-CRY1 or FHM-CRY2 were treated with 10 μM MG132 for 6 hours and lysed with IP Buffer. Cell lysates were subjected to immunoprecipitation using anti-FLAG-M2 agarose beads. FH-LacZ expressed in NIH3T3 cells was used as a control. **B**. The numbers of proteins co-purified with FHM-CRY1 or FHM-CRY2. Proteins co-purified with FH-LacZ were eliminated from the list of CRY1 and CRY2 interacting proteins. Proteins detected in both CRY1 and CRY2 samples with high MS scores were listed in S1 Table. **C.** Interaction of USP7 with CRY2 protein. NIH3T3 cells expressing FLAG-CRY2 and/or Myc-USP7 were cultured in the presence of 10 μM MG132 for 6 hours and lysed with IP Buffer. The cell lysates were subjected to immunoprecipitation using anti-FLAG, anti-Myc antibody or normal mouse IgG (negative control) as precipitating antibodies.

To explore regulators of the protein stabilities of CRY1 and CRY2, we performed CRY interactome analysis using highly sensitive LC-MS/MS-based shotgun proteomics. FLAG-tagged CRY1 or CRY2 was affinity-purified from NIH3T3 cells, and 216 proteins were detected as CRY-interacting proteins (Fig 1A and 1B and S1–S4 Tables). The proteins identified as interacting with both CRY1 and CRY2 included FBXL3, SKP1, CKIδ, glucocorticoid receptor (GR) and DDB1, which were previously reported to bind with CRY1 or CRY2 [12,13,22–24]. The interaction of CRY with TRIM28, KCTD5 and DDB1 was confirmed by co-immunoprecipitation assay (S1 Fig). Among these proteins, we found USP7, a deubiquitinating enzyme which is also known as a herpesvirus-associated ubiquitin-specific protease (HAUSP) [18]. USP7 is involved in regulation of p53 and its E3 ligase, Mdm2, through their deubiquitination [25]. We also verified the interaction of Myc-USP7 with FLAG-CRY2 in NIH3T3 cells by co-immunoprecipitation assay. Myc-USP7 was co-immunoprecipitated with FLAG-CRY2, and similarly FLAG-CRY2 was co-immunoprecipitated with Myc-USP7 (Fig 1B).

**Fig 2:**
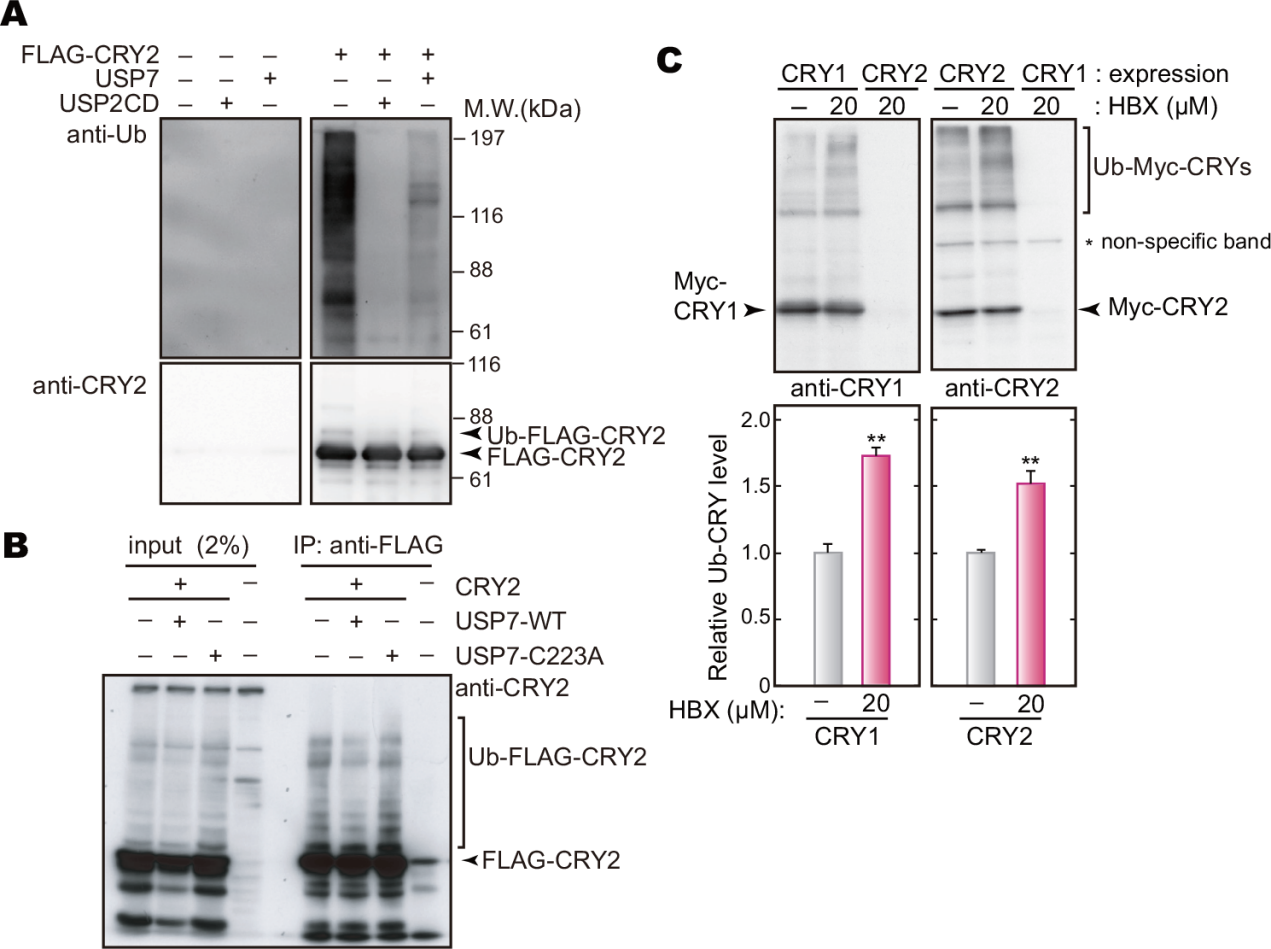
USP7 deubiquitinates CRY proteins. **A**. *In vitro* ubiquitination assay. HEK293T/17 cells were transfected with the expression vector of FLAG-CRY2. Forty-two hours after the transfection, the cells were cultured in the presence of 10 μM MG132 for 6 hours and then harvested. FLAG-CRY2 purified from the cell lysate with anti-FLAG M2 agarose beads was incubated with or without a recombinant protein, full-length USP7 or a catalytic domain of USP2 (USP2 CD), for 30 min at 37°C. Recombinant USP2 catalytic domain was used as a positive control [26]. **B**. *In vivo* deubiquitination assay in HEK293T/17 cells. The cells were transfected with indicated expression vectors. Forty-two hours after the transfection, the cells were cultured in the presence of 10 μM MG132 for 6 hours and then lysed with IP Buffer. FLAG-CRY2 was purified with anti-FLAG M2 agarose beads, followed by western blotting analysis with anti-CRY2 antibody. An inactive mutant of USP7 (USP7-C223A) was used for a negative control. **C.** Effect of USP7-specific inhibitor on CRY up-shifted bands. NIH3T3 cells were transfected with the expression vector for Myc-CRY1 or Myc-CRY2. Forty-two hours after the transfection, the cells were cultured in the presence of 20 μM HBX 41108 for 6 hours. The smear bands of Myc-CRY1 or Myc-CRY2 were quantified (means + SEM, n = 3, **: *p* < 0.01 by Student’s *t*-test).

We then asked whether CRY is a substrate of USP7-catalyzed deubiquitination by *in vitro* deubiquitination assay. As a positive control, recombinant protein USP2 catalytic domain [26] was incubated with FLAG-CRY2 purified from NIH3T3 cells, by which the up-shifted smear bands of FLAG-CRY2 were reduced (Fig 2A). Under these conditions, incubation of FLAG-CRY2 with USP7 similarly decreased the smear bands of CRY2 (Fig 2A). We examined deubiquitinating activity of USP7 on CRY2 in cultured HEK293T/17 cells by *in vivo* deubiquitination assay. Co-expression of Myc-USP7 decreased up-shifted form of FLAG-CRY2 (Fig 2B), whereas co-expression of catalytically inactive mutant, USP7-C223A [27], caused no detectable change in the band densities (Fig 2B). Similar results were seen in pharmacological analysis, in which the up-shifted bands of Myc-CRY1 and Myc-CRY2 expressed in NIH3T3 cells were significantly increased by treatment with USP7-specific inhibitor, HBX 41108 [28](Fig 2C). These results demonstrate that USP7 deubiquitinates CRY proteins.

### USP7 stabilizes CRY proteins

**Fig 3:**
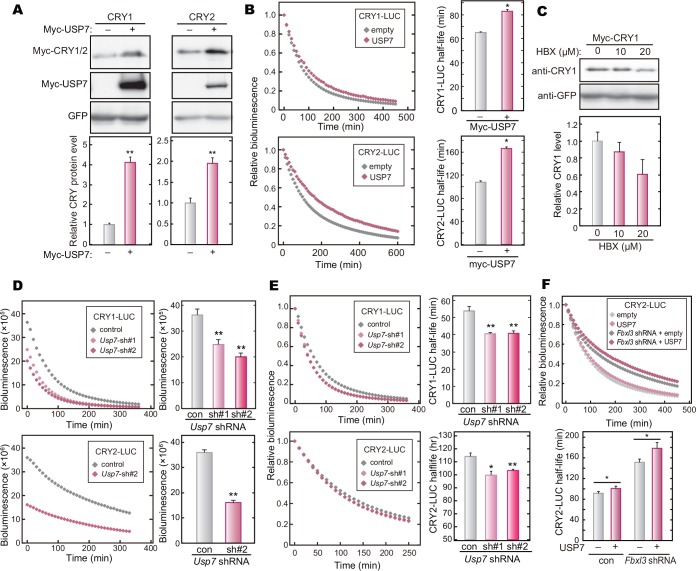
USP7 increases the protein levels and stabilities of CRY1 and CRY2. **A.** Effect of USP7 expression on CRY protein levels. HEK293T/17 cells were transfected with indicated expression vectors. Forty-eight hours after the transfection, the cells were lysed with SDS-PAGE sample buffer, and the cell lysate was analyzed by western blotting. GFP was used as a control. Quantified data are shown by means + SEM (n = 3, **: *p* < 0.01 by Student’s *t*-test). **B.** Effect of USP7 overexpression on CRY-LUC protein stability. HEK293T/17 cells were transfected with expression vectors for CRY-LUC and Myc-USP7 (or empty control), and cultured for 48 hours. The culture medium was changed to the recording medium containing 0.1 mg/ml cycloheximide. Bioluminescence signals were recorded continuously at 10-min intervals, and the signal was normalized to the value at time 0. Half-lives of CRY1-LUC and CRY2-LUC were calculated by fitting an exponential decay curve to bioluminescence signals, and are shown as means + SEM (n = 3, **: *p* < 0.01 by Student’s *t*-test). **C.** USP7 inhibitor treatment of NIH3T3 cells decreased Myc-CRY1 expression. NIH3T3 cells were transfected with Myc-CRY1 and GFP expression vectors. Forty-two hours after the transfection, the cell were cultured with 10 or 20 μM HBX 41108 for 6 hours. Quantified data are shown as means + SEM (n = 3). **D.** *Usp7* knockdown decreased the starting levels of CRYs-LUC. The decay of the bioluminescence signals was recorded as described in **B**. The starting levels of CRYs-LUC bioluminescence signals are shown as means + SEM (n = 4, **: *p* < 0.01 by Student’s *t*-test). **E.** *Usp7* knockdown decreased CRYs-LUC stability. The bioluminescence signals normalized to the value at time 0 and the half-lives of CRYs-LUC are shown as means + SEM (n = 4, **: *p* < 0.01 by Tukey’s test or Student’s *t*-test). **F.** Effect of USP7 overexpression on CRY2-LUC protein stability in *Fbxl3* knockdown cells. HEK293T/17 cells were transfected with indicated plasmid vectors and cultured for 72 hours. The decay of the bioluminescence signals was recorded as described in **B**. Quantified data are shown as means + SEM (n = 3, *: *p* < 0.05 by Student’s *t*-test).

In general, deubiquitinating enzymes including USP family proteins edit free ubiquitin chains or ubiquitin(s) attached to proteins, modulating the protein function by regulating stability, localization and signal transduction [29]. We previously showed that ubiquitination of CRYs by FBXL3 and FBXL21 promotes their proteasomal degradation and stabilization, respectively, presumably by catalyzing formation of ubiquitin chains with linkage modes different from each other [15]. Furthermore, it was recently reported that DDB1 and FBXW7 also ubiquitinate CRYs and promote the proteasomal degradation [24,30]. Thus, it is likely that USP7 regulates the protein stabilities of CRYs by cleaving the ubiquitin chains attached by these E3 ligases. To explore this possibility, we examined the effect of overexpression of USP7 on CRY stabilities. The protein levels of Myc-CRY1 and Myc-CRY2 were increased by co-expression of Myc-USP7 in HEK293T/17 cells (Fig 3A). The protein levels of Myc-PER2 (S2 Fig) and GFP (Fig 3A) were unaffected by overexpression of Myc-USP7, indicating that CRY1 and CRY2 are the specific targets of USP7. We then determined the effect of USP7 on the lifetimes of CRY1-LUC and CRY2-LUC proteins by recording the decay rates of their bioluminescence signals in HEK293T/17 cells [15,31]. Overexpression of Myc-USP7 significantly lengthened the lifetimes of CRYs-LUC (Fig 3B), while having no significant effect on the lifetime of LUC protein (S3 Fig).

In contrast to USP7 overexpression, treatment of HEK293T/17 cells with HBX 41108 reduced the steady state levels of Myc-CRY1 protein in a dose-dependent manner (Fig 3C). HBX 41108 treatment also accelerated CRY2-LUC degradation in HEK293T/17 cells (S4 Fig). We then knocked down *Usp7* by shRNA in HEK293T/17 cells (S5A and S5B Fig). The starting levels of the bioluminescence signals derived from CRY1-LUC or CRY2-LUC in *Usp7*-knockdown cells were far lower than that in the control cells when the recording started (with the addition of cycloheximide) 72 hours after the transfection (Fig 3D). This observation indicated that *Usp7* knockdown decreased the steady state levels of CRY1 and CRY2 proteins. The half-lives of CRY1-LUC and CRY2-LUC in *Usp7*-knockdown cells were significantly shorter than those in the control cells, while there was no discernable effect of *Usp7* knockdown on LUC stability (Fig 3E and S5C Fig). To ask whether USP7 specifically inhibits CRY degradation promoted by FBXL3, we examined USP7-mediated stabilization of CRY in *Fbxl3*-knockdown cells (S5D Fig). *Fbxl3* knockdown in HEK293T/17 cells did not abrogate USP7-dependent stabilizing effect on CRY2 (Fig 3F), suggesting that USP7 deubiquitinates CRY protein that was ubiquitinated by multiple E3 ligases. Together, we concluded that USP7 stabilizes CRY proteins and consequently regulates CRY levels in the cultured cells.

### Inhibition of USP7 shortens the circadian period

**Fig 4:**
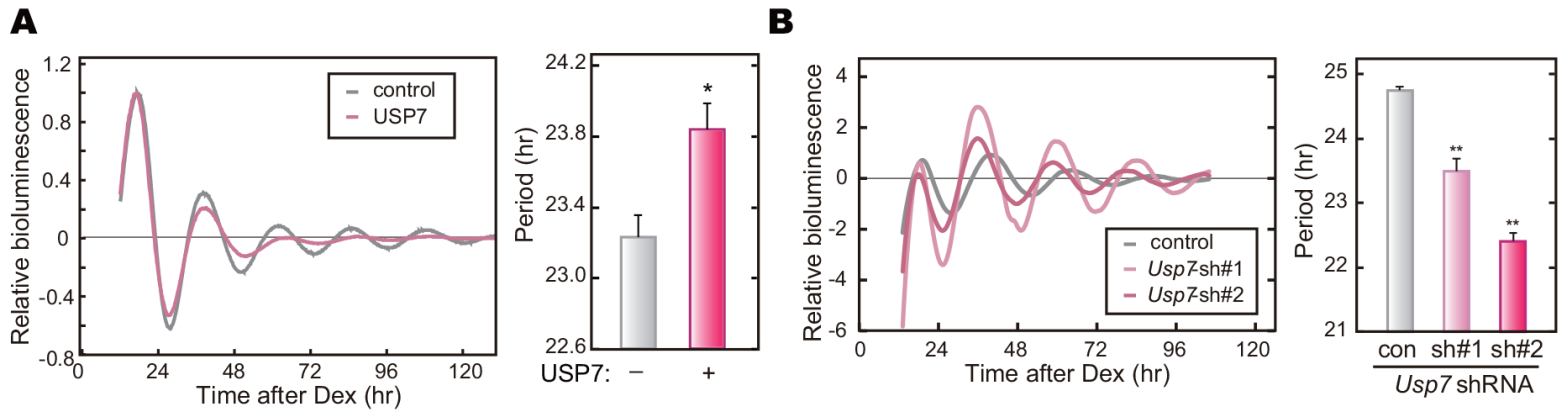
*Usp7* knockdown shortens the circadian period of the cellular rhythms. **A.** NIH3T3 cells were transfected with a luciferase reporter vector, *Bmal1* us0.3-luc, and USP7 expression vector. Twenty-four hours after the transfection, the cellular rhythms were synchronized by 30-min treatment with 0.1 μM dexamethasone (Dex). The culture medium was changed to the recording medium, and bioluminescence signals were recorded continuously. The calculated circadian periods are shown as means + SEM (n = 4, *: *p* < 0.05 by Student’s *t*-test). **B.** Cellular rhythms in *Usp7* knockdown cells. Bioluminescence rhythms of *Bmal1*-luc reporter were recorded as described in **A**. The calculated circadian periods are shown as means + SEM (n = 4, **: *p* < 0.01 by Tukey’s test).

Previous studies demonstrated that dysregulation of CRY stabilities caused abnormal periodicity in mouse behavioral rhythms and gene expression rhythms [12,14,15,17,31,32]. For example, knockdown or knockout of *Fbxl3* lengthened the circadian period [15,16], whereas knockdown or mutation of *Fbxl21* shortened the period [15,17]. Here we found that overexpression of USP7 lengthened the circadian period of the cellular rhythms (Fig 4A). In contrast, treatment of PER2::LUC mouse embryonic fibroblast (MEF) with HBX 41108 shortened the circadian period (S6A Fig). Similar to the inhibitor treatment, shRNA-mediated *Usp7* knockdown resulted in shortening of the circadian period in NIH3T3 cells (Fig 4B and S6B Fig). These results are consistent with the previous findings that stabilization of CRY proteins lengthened the period of the circadian rhythms and destabilization of CRYs by *Fbxl21* knockdown caused a shortening of the period [11].

### TDP-43 stabilizes CRY proteins and regulates the circadian period

**Fig 5:**
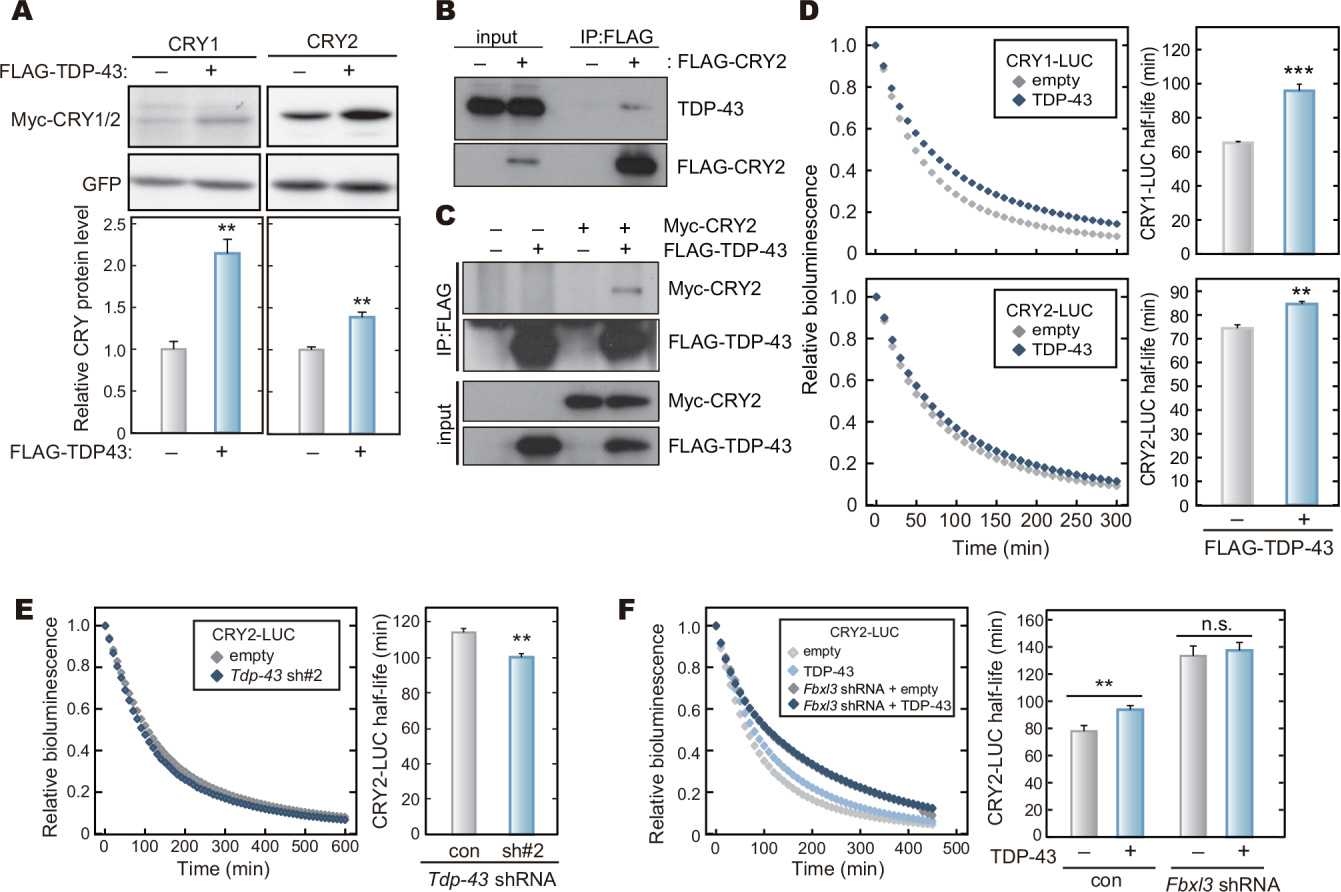
TDP-43 interacts with CRY proteins and stabilizes CRY proteins. **A.** Effect of TDP-43 expression on CRY protein levels. HEK293T/17 cells were transfected with expression vectors for Myc-CRY1, Myc-CRY2 and FLAG-TDP-43, and cultured for 48 hours. The cells were lysed with SDS-PAGE sample buffer, and the cell lysate was analyzed by western blotting. Quantified data are shown by means + SEM (n = 3, **: *p* < 0.01 by Student’s *t*-test). GFP was used as a control. **B, C.** Interaction of TDP-43 with CRY2 protein. HEK293 cells expressing FLAG-CRY2 were lysed with IP buffer, followed by immunoprecipitation using anti-FLAG antibody. Binding of endogenous TDP-43 with FLAG-CRY2 was detected by western blotting analysis (**B**). HEK293 cells expressing Myc-CRY2 and/or FLAG-TDP-43 were lysed with IP Buffer. The cell lysates were subjected to immunoprecipitation using anti-FLAG antibody (**C**). **D.** Effect of TDP-43 overexpression on CRY-LUC stability. HEK293T/17 cells were transfected with CRYs-LUC and TDP-43 expression vectors and cultured for 48 hours. The culture medium was changed to the recording medium containing 0.1 mg/ml cycloheximide and the bioluminescence signals of CRY-LUC were recorded. Bioluminescence signal at the start time point was set to 1. Half-lives of CRY1-LUC and CRY2-LUC were calculated by fitting an exponential decay curve to bioluminescence signals and shown as means + SEM (n = 4, **: *p* < 0.01, ***: *p* < 0.001 by Student’s *t*-test). **E.** *Tdp-43* knockdown decreased CRY2-LUC stability. The decay of the bioluminescence signals of CRY2-LUC was recorded, and the half-life of CRY2-LUC was calculated as described in **D**. Quantified data are shown as means + SEM (n = 4, **: *p* < 0.01 by Student’s *t*-test). **F.** Effect of TDP-43 overexpression on CRY2-LUC protein stability in *Fbxl3*-knockdown cells. HEK293T/17 cells were transfected with indicated expression vectors and cultured for 72 hours. The decay of the bioluminescence signals was recorded as described in **D**. Quantified data are shown as means + SEM (n = 4, **: *p* < 0.01 by Student’s *t*-test).

In the screening for regulators of CRYs stabilities, we paid special attention to not only ubiquitination-related enzymes but also other CRY-interacting proteins with high MS scores, which was an indicator of peptide quantity and quality identified by MS/MS analysis (S1 Table). Among these proteins, we found that TDP-43 increased the protein amount of CRY1 or CRY2 when co-expressed in HEK293T/17 cells (Fig 5A). TDP-43 protein is an RNA-binding protein and it is responsible for the onset of neuronal diseases, amyotrophic lateral sclerosis (ALS) and frontotemporal lobar degeneration (FTLD). TDP-43 binds to target mRNAs such as *Neurofilament* (*Nefl*) and *Survival of motor neuron 2* (*Smn2*), and regulates the mRNA stability, splicing, translocation, and translation [20,33]. Nevertheless, TDP-43 was included in the present CRY1 and CRY2 interactome (S1 Table), and we confirmed their protein-protein interaction by co-immunoprecipitation assay (Fig 5B and 5C). To ask whether TDP-43 regulates CRY protein stability through the protein-protein interaction, we examined the degradation rate of CRY1-LUC or CRY2-LUC co-expressed with TDP-43. Overexpression of FLAG-TDP-43 lengthened the half-lives of CRY1-LUC and CRY2-LUC in HEK293T/17 cells (Fig 5D), while FLAG-TDP-43 had no effect on the stability of LUC control (S7 Fig). On the other hand, knockdown of *Tdp-43* by shRNA accelerated degradation of CRY2-LUC (Fig 5E and S8 Fig). In contrast to the action of USP7, the stabilizing effect of TDP-43 overexpression on CRY2 protein was abrogated in *Fbxl3* knockdown cells (Fig 5F), suggesting that TDP-43 specifically protects CRY from FBXL3-mediated degradation by interfering with FBXL3 function. We concluded that TDP-43 stabilizes CRY1 and CRY2 proteins and increases their expression levels.

**Fig 6:**
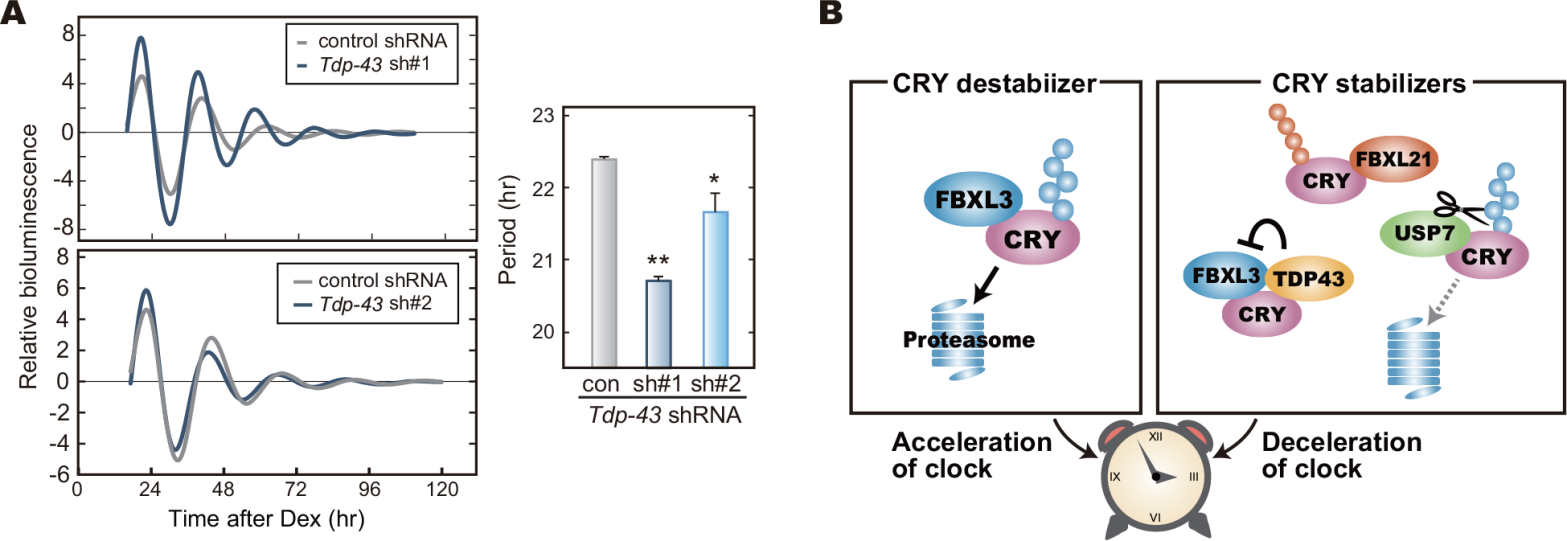
*Tdp-43* knockdown shortens the circadian period. **A.** Cellular rhythms in *Tdp-43* knockdown cells. Bioluminescence rhythms of *Bmal1*-luc reporter were recorded as described in Fig 4A. Calculated periods of the cellular rhythms are shown as means + SEM (n = 4, *: *p* < 0.05, **: *p* < 0.01 by Student’s *t*-test). **B.** A model for period-determination by the control of CRY proteins stabilities. CRY protein destabilizer, FBXL3, accelerates the oscillation of the circadian clock, while CRY stabilizers slow down the oscillation speed.

We then determined the effect of *Tdp-43* knockdown on the cellular rhythms of NIH3T3 cells. We found that *Tdp-43* knockdown shortened the circadian period (Fig 6A). These results (Figs 4 and 6A) raised a model, in which destabilization of CRY proteins shortens the circadian period of the cellular rhythms (Fig 6B), and emphasize the important role of the protein stabilities of CRY1 and CRY2 for period determination of the circadian clock.

## Discussion

In many species, ubiquitination of clock proteins has been reported to play multiple roles in their clockwork [11,34]. Of note, ubiquitination mediated by ubiquitin E3 ligases is a reversible reaction, which is catalyzed by deubiquitinating enzymes [35]. The ubiquitin editing events such as cleavage and remodeling of the ubiquitin chains are important processes for ubiquitination-mediated cellular functions. However, studies on deubiquitinating enzymes have been left behind in the research field of chronobiology [11]. It has been recently reported that BMAL1, PER1 and CRY1 are deubiquitinated by Ubiquitin-specific protease 2 (USP2) in mammals, and that *Usp2* knockout mice exhibited altered response to light in the behavioral rhythms [26,36,37]. USP17 was reported to control DNA-damage response by stabilizing one of the clock proteins, DEC1 [38], while the role of USP17 in the clockwork remains elusive. In the present study, we identified USP7 as a deubiquitinating enzyme of CRY1 and CRY2 in screening of the CRY interactome (Fig 1). Very recently, it has been shown that CRY1 interacts with USP7 when cultured cells respond to DNA damage and that USP7 protects CRY1 from FBXL3-mediated degradation [19]. They showed a significant contribution of USP7 to CRY1 stability controlled by deubiquitination in proper response to DNA-damage for DNA repair [19]. Here, we demonstrated that, even in the absence of any DNA damage-inducible stimuli, not only CRY1 but also CRY2 binds to USP7 and is deubiquitinated by USP7 (Figs 1 and 2). USP7 plays an important role in regulating stabilities of both CRY1 and CRY2 for maintaining the normal period of the circadian clock oscillation (Fig 4).

We previously reported that FBXL3 and FBXL21 catalyze formation of different types of ubiquitin chains at different lysine residues of CRY proteins, leading to degradation and stabilization of CRYs, respectively [15]. USP7 stabilizes CRYs and *Usp7* knockdown shortened the circadian periods (Figs 3 and 4B), implying that USP7 removes ubiquitin chain(s) acting as a proteasomal degradation signal. However, it is still possible that USP7 may antagonize the action of FBXL21 by cleaving the ubiquitin chain(s) formed by FBXL21. It should be emphasized that almost all the members of USP family appear to have no particular preference to any specific chain linkages to cleave [35]. Furthermore, a recent study of USP7 protein structure indicated that it could cleave any types of ubiquitin chains [39]. Recently, two research groups reported that, besides FBXL3, FBXW7 and DDB1 also promote CRY degradation through ubiquitination [24,30]. Hence it is likely that USP7 cleaves ubiquitin chains generated by multiple E3 ligases, and consequently governs the stabilities of CRY1 and CRY2. This idea is supported by the observation that solo knockdown of *Fbxl3* did not mask the stabilizing effect of USP7 on CRY2 (Fig 3F). On the other hand, TDP-43 stabilizes CRY2 in a manner dependent on FBXL3 (Fig 5F), suggesting that TDP-43 interferes with FBXL3 function probably by binding to CRY proteins.

TDP-43 has been previously characterized as an RNA-binding protein and the biological significance of TDP-43 in RNA metabolism has been well established [20,40]. Intriguingly, hyper-phosphorylated/ubiquitinated TDP-43 is also a major pathological protein causing neurodegenerative diseases, such as ALS and FTLD [20,40]. In patients with these diseases, disturbed hormonal rhythms and sleep disorders are frequently observed [41,42]. Our findings may provide a new link between the circadian clock and these neurological pathologies. In the present study, we demonstrated a noticeable role of TDP-43 in regulating CRY stabilities probably through protein-protein interaction (Fig 5). Regulation of the clock(-related) protein stability by an RNA-binding protein was previously found in *Neurospora* circadian clockwork [43,44], in which FRQ-interacting RNA helicase (FRH) binds to FRQ, a principal repressor of the negative feedback loop [43]. FRH binding to *frq* mRNA helps protein folding of FRQ immediately after its translation, and protects FRQ from proteasomal degradation [44]. Interestingly, while RNA helicase activity of FRH is not essential for the clock function, FRH-FRQ interaction is necessary for FRQ stabilization and the circadian oscillation [44]. To our knowledge, the present work is the first report that the protein stabilization mediated by an RNA-binding protein is important for the mammalian circadian clock, while the molecular mechanism underlying the CRY stabilization remains to be elucidated.

Previous studies reported that *Fbxl3* mutant mice or *Fbxl3* null mice showed the behavioral and cellular rhythms with extremely long circadian periods [12,14,16]. Conversely, *Fbxl21* knockdown or its mutation caused short periods [15,17]. Thus, FBXL3 acts as a destabilizer of CRY1 and CRY2 to accelerate the circadian oscillation of the molecular clock, whereas FBXL21 is a stabilizer of CRYs to decelerate the clock oscillation (Fig 6B). In the present study, we demonstrated that USP7 and TDP-43 are the stabilizers of both CRY proteins and that knockdown of *Usp7* or *Tdp-43* shortened the circadian period. A general principle appears to participate in the mechanism, by which the fine-tuning of CRY protein stability controls the circadian period (Fig 6B) and this idea is consistent with a simulation study predicting that the protein degradation rate of CRY is a critical determinant of the circadian period [45]. Collectively, we conclude that a complex network of regulation for the protein stabilities of CRY proteins contributes to the robust and stable oscillation of the circadian clock.

## Material and Methods

### Cell culture and plasmids for transfection

NIH3T3 (Riken Cell Bank) and HEK293T/17 cells were cultured and passaged under 5% CO_2_ in DMEM (Sigma Aldrich), 100 U/ml penicillin, 100 μg/ml streptomycin, and 10% fetal bovine serum (Equitech Bio). Mammalian expression vectors of pCMV-Tag 3B-Myc-mCRY1, pCMV-Tag 3B-Myc-mCRY2, pcDNA3.1-FLAG-His-Myc-mCRY1 (FHM-CRY1), pcDNA3.1-FLAG-His-Myc-mCRY2 (FHM-CRY2) and pcDNA3.1-FLAG-His-LacZ (FH-LacZ) were constructed as previously described [10,15]. For mammalian expression vectors of pcDNA3.1-FLAG-mTDP-43 and pcDNA3.1-FLAG-hFBXL3, full length mouse *Tdp-43* or human *Fbxl3* CDS with an oligonucleotide encoding FLAG epitope sequence was cloned into pcDNA3.1. Expression plasmids of pCS2-6×Myc-mPER2 and pcDNA3-6×Myc-hUSP7 were kindly provided by Dr. Louis Ptacek (University of California, San Francisco) and Dr. Akiko Murayama (University of Tsukuba), respectively. Reporter vectors used for degradation assay, p3×FLAG-CMV14-mCRY1-LUC, p3×FLAG-CMV14-mCRY2-LUC and p3×FLAG-CMV14- LUC [31], were kindly provided by Dr. Steve A. Kay and Dr. Tsuyoshi Hirota. The catalytic inactive form of human USP7 expression vector (pcDNA3-6×Myc-hUSP7-C223A) was created by site-direct mutagenesis. shRNA vectors targeting *Usp7* or *Tdp-43* were generated by inserting target sequences into the pSilencer3.1-H1 puro vector (Ambion). Target sequences were 5'-GTGTG AAATT CCTAA CATTG C-3' (human and mouse *Usp7* sh1), 5'-GTCCC TTTAG CATTA CAAAG A-3' (human and mouse *Usp7* sh2), 5'-GTAGA TGTCT TCATT CCCAA A-3' (mouse *Tdp-43* sh1), and GCAAT AGACA GTTAG AAAGA A-3' (human and mouse *Tdp-43* sh2), 5'-CGGCC ACTTG ATGAA GAGTT A-3' (human *Fbxl3*). As a control, scrambled sequence was used as previously described [46]. pEGFP-C1 (Clontech) was used for transfection and loading control. pcDNA3, pcDNA3.1 and pSilencer3.1-H1 puro vector were used for negative (empty vector) controls for USP7, TDP-43 expression and knockdown experiments, respectively.

### Antibodies and reagents

Anti-CRY1 or anti-CRY2 polyclonal antibody was raised in rabbits by using partial fragments of mouse CRY1 (506–606) or mouse CRY2 (524–592) as antigen peptides, respectively [15]. Other antibodies were obtained from the following commercial vendors: anti-USP7 (Bethyl Laboratories), anti-Myc, anti-ubiquitin, anti-GFP (Santa Cruz Biotechnology) and anti-FLAG (Sigma Aldrich). Transfection into the cultured cells was performed with Lipofectamine 2000 reagent (Life Technologies) or polyethylenimine (Polysciences) with standard protocols. USP7 specific inhibitor HBX 41108 was purchased from Boston Biochem and solved in DMSO, which was used as a vehicle control of the inhibitor treatment. Silver stain MS kit (WAKO) was used for silver staining of gels according to the manual.

### MS spectrometry analysis

Proteins purification and shotgun proteomic analysis were performed as previously described [15]. Briefly, NIH3T3 cells were transfected with expression vectors for FLAG-His-Myc-CRY1, FLAG-His-Myc-CRY2 or FLAG-His-LacZ (as a control). FLAG-tagged proteins were immunoprecipitated by FLAG-M2 agarose affinity gel (Sigma Aldrich). LacZ or CRY proteins were eluted by 150 ng/μl FLAG-peptide (Sigma Aldrich) followed by tryptic digestion. Shotgun proteomic analyses were performed by a linear ion trap-orbitrap mass spectrometer (LTQ-Orbitrap Velos, Thermo Fisher Scientific) coupled with nanoflow LC system (Dina-2A, KYA Technologies).

### Co-immunoprecipitation

Transfected NIH 3T3 cells were lysed for 30 min in ice-chilled IP Buffer [20 mM HEPES-NaOH, 137 mM NaCl, 2 mM EDTA, 10% (v/v) glycerol, 1% (v/v) Triton X-100, 1 mM DTT, 4 μg/ml aprotinin, 4 μg/ml leupeptin, 50 mM NaF, 1mM Na_3_VO_4_, 1 mM phenylmethylsulfonyl fluoride; pH 7.8]. The lysate was incubated with a precipitating antibody solution for 2 hr at 4°C, followed by incubation with 20 μl Protein G-Sepharose beads for 1 hr at 4°C. The beads were washed three times with IP Buffer and then subjected to immunoblotting.

### Western blotting

Proteins separated by SDS-PAGE were transferred to polyvinylidene difluoride membrane (Millipore). The blot membranes were blocked in a blocking solution (1% [w/v] skim milk in TBS [50 mM Tris-HCl, 140 mM NaCl, 1 mM MgCl_2_; pH 7.4]) for 1 hr at 37°C, and then incubated overnight at 4°C with a primary antibody in the blocking solution. The signals were visualized by an enhanced chemiluminescence detection system (PerkinElmer Life Science). The blot membrane was subjected to densitometric scanning and the band intensities were quantified by using ImageJ software v.10.2.

### *In vitro* deubiquitination assay

HEK293T/17 cells expressing FLAG-CRY2 proteins were treated with 10 μM MG132 for 6 hr and then lysed in IP Buffer for 30 min. The cell lysate was incubated with 20 μl of anti-FLAG M2 beads (Sigma Aldrich) for 2 hr at 4°C. FLAG-CRY2 was eluted with 150 μg/ml FLAG epitope peptide solution (Sigma Aldrich). Eluted FLAG-CRY2 was incubated with human recombinant protein of USP2 catalytic domain (Life Sensors) or human USP7 (Life Sensors) in USP reaction buffer (10 mM NaH_2_PO_4_, 140 mM NaCl, 5 mM MgCl_2_, 2 mM DTT; pH 7.4) for 30 min at 37°C, followed by western blotting analysis.

### *In vivo* deubiquitination assay

FLAG-CRY2 proteins were co-expressed with Myc-USP7 or with Myc-USP7-C223A in HEK293T/17 cells. The cells were treated with 10 μM MG132 for 6 hr and then lysed for 30 min in IP Buffer. The cell lysate was incubated with 1 μg of anti-FLAG M2 antibody (Sigma Aldrich) for 2 hr at 4°C, followed by incubation with 20 μl Protein G-Sepharose beads for 1 hr at 4°C. The beads were washed three times with IP Buffer and then subjected to immunoblotting.

### CRY-LUC degradation assay

HEK293T/17 cells were transfected with an expression vector for CRY1-LUC, CRY2-LUC or LUC (control) [31] and cultured for 24 or 48 hours. The cultured medium was changed to the recording medium (described in the method of Real-time monitoring of rhythmic gene expression) containing cycloheximide (Nacalai Tesque; 100 μg/ml in final concentration). Luciferase activity of CRY-LUC was recorded at 10-min intervals at 37°C in air with Dish Type Luminescencer, Kronos (ATTO) or LumicCycle 32 (Actimetrics). The half-life of CRY-LUC was estimate by fitting an exponential decay curve to normalized bioluminescence signals. More detailed experimental conditions were described in Supporting Information (S5 Table).

### Real-time monitoring of rhythmic gene expression

Real-time monitoring of the luciferase expression rhythm was performed as previously described [46] with a minor modification to the recording medium: phenol-red free DMEM (Sigma Aldrich) supplemented with 10% fetal bovine serum (Equitech Bio, Inc.), 3.5 mg/ml glucose, 25 U/ml penicillin, 25 μg/ml streptomycin, 0.1 mM luciferin, and 10 mM HEPES-NaOH (pH 7.0).

## Supporting Information

S1 Fig Interaction of identified proteins with CRY1. **A.** Interaction of TRIM28 with CRY1. HEK293T/17 cells expressing Myc-CRY1 and/or FLAG-TRIM28 were cultured in the presence of 10 μM MG132 for 6 hours and lysed with IP Buffer. The cell lysates were subjected to immunoprecipitation using anti-Myc (left panel), anti-FLAG (right) antibody, or normal mouse IgG (negative control) as precipitating antibodies. **B.** Interaction of KCTD5 with CRY1 protein. Co-immunoprecipitation was performed as described in **A**. **C.** Interaction of DDB1 with CRY1 or CRY2 proteins. Co-immunoprecipitation was performed as described in **A**.
(EPS)

S2 Fig Effect of Myc-USP7 overexpression on Myc-PER2 protein levels. HEK293T/17 cells were transfected with indicated expression vectors and cultured for 48 hours. Then the cells were lysed with SDS-PAGE sample buffer, and the lysate was analyzed by Western blotting. GFP was used for transfection and loading controls. Quantified data were shown by means + SEM (n = 3). n.s. represents non-significant change (p > 0.05 by Student’s *t*-test).
(EPS)

S3 Fig Effect of Myc-USP7 overexpression on LUC stability. HEK293T/17 cells were transfected with expression vectors for LUC and Myc-USP7 (or the empty vector) and cultured for 48 hours. The culture medium was changed to the recording medium containing 0.1 mg/ml cycloheximide. Bioluminescence signals were recorded continuously at 10-min intervals and normalized to the value at time 0. Half-life of LUC was calculated by fitting an exponential decay curve to the bioluminescence signals and shown as means + SEM (n = 3).
(EPS)

S4 Fig Effect of USP7 inhibitor on CRY2-LUC stability. HEK293T/17 cells were transfected with expression vectors for CRY2-LUC and cultured for 24 hours. The culture medium was changed to the recording medium containing 20 μM HBX 41108 and 0.1 mg/ml cycloheximide. Bioluminescence signals were recorded continuously at 10-min intervals and normalized to the value at time 0.
(EPS)

S5 Fig Effect of *Usp7* knockdown on LUC stability. **A.** HEK293T/17 cells were transfected with Myc-USP7 and shRNA vectors targeting human *Usp7*, and cultured for 72 hours. The cells were lysed with SDS-PAGE sample buffer, and the cell lysate was analyzed by western blotting. The band intensities of Myc-USP7 were normalized to GFP levels. The quantified data are shown as means + SEM (n = 3). **B.** Knockdown of endogenous USP7 in HEK293T/17 cells. HEK293T/17 cells were transfected with the shRNA expressing vectors, and the transfected cells were selected by puromycin. **C.** HEK293T/17 cells were transfected with the shRNA expressing vectors. The cells were cultured for 72 hours, and the culture medium was changed to the recording medium containing 0.1 mg/ml cycloheximide. Bioluminescence signals were recorded continuously at 10-min intervals and normalized to the value at time 0 (upper panel). Half-life of LUC was calculated by fitting an exponential decay curve to the bioluminescence signals (control: 105 +/- 12 min, shRNA#1: 112 +/- 12 min, shRNA#2: 94 +/- 10 min) and shown as means + SEM (n = 3, lower left panel). The starting levels of CRYs-LUC bioluminescence signals were shown as means + SEM (n = 3, lower right panel). n.s. represents non-significant change (p>0.05 by Tukey’s test). **D.** HEK293T/17 cells were transfected with FLAG-FBXL3 and *Fbxl3* shRNA expression vectors, and cultured for 72 hours. The cells were then lysed with SDS-PAGE sample buffer, and the cell lysate was analyzed by western blotting. The band intensities of FLAG-FBXL3 were normalized to GFP levels, and shown as means + SEM (n = 3).
(EPS)

S6 Fig Bioluminescence rhythms of the cultured cells treated with USP7 inhibitor and knockdown efficiency of *Usp7*. **A.** The cellular rhythms of PER2::LUC MEF were synchronized by 30-min treatment of 0.1 μM dexamethasone (Dex). The culture medium was changed to the recording medium including HBX 41108, and the bioluminescence signals of PER2::LUC were recorded continuously (left panel). The calculated period lengths are shown as means + SEM (n = 4, right panel). **B.** NIH3T3 cells were transfected with Myc-USP7 and *Usp7*-targeting shRNA expression vectors, and cultured for 72 hours. The cells were lysed with SDS-PAGE sample buffer, and the cell lysate was analyzed by western blotting. The band intensities of Myc-USP7 were normalized to β-actin levels and the quantified data are shown as means + SEM (n = 3).
(EPS)

S7 Fig Effect of TDP-43 protein expression on LUC stability. HEK293T/17 cells were transfected with expression vectors for LUC and FLAG-TDP-43. The cells were cultured for 48 hours, and the culture medium was changed to the recording medium containing 0.1 mg/ml cycloheximide. Bioluminescence signals were recorded continuously at 10-min intervals and normalized to the value at time 0. Half-lives of LUC were calculated by fitting exponential decay curves to the bioluminescence signals and shown as means + SEM (n = 4). n.s. represents non-significant change (p>0.05 by Student’s *t*-test)
(EPS)

S8 Fig Knockdown efficiency of *Tdp-43*. **A.** HEK293T/17 cells were transfected with expression vectors for FLAG-TDP-43 and shRNA targeting *Tdp-43*, and cultured for 72 hours. The cells were lysed with SDS-PAGE sample buffer, and the cell lysate was analyzed by Western blotting. The band intensities of FLAG-TDP-43 were normalized to GFP levels and averaged data are shown as means + SEM (n = 3). **B.** Knockdown of endogenous TDP-43 in NIH3T3 cells. NIH3T3 cells were transfected with shRNA expression vectors targeting *Tdp-43*, and the transfected cells were selected by puromycin.
(EPS)

S1 Table Proteins co-purified with either CRY1 or CRY2. Proteins with Mascot score above 100 in CRY1 or CRY2 interactome analysis were listed.
(DOCX)

S2 Table List of peptides identified in LacZ sample. (XLSX)

S3 Table List of peptides identified in CRY1 sample. (XLSX)

S4 Table List of peptides identified in CRY2 sample. (XLSX)

S5 Table Detailed transfection conditions for the degradation assay. (DOCX)

## References

[1] Takahashi JS. Molecular neurobiology and genetics of circadian rhythms in mammals. Annu Rev Neurosci. 1995; 18: 531–553. 10.1146/annurev.ne.18.030195.002531 7605073

[2] Reppert SM, Weaver DR. MOLECULAR ANALYSIS OF MAMMALIAN CIRCADIAN RHYTHMS. Annu Rev Physiol. 2001; 63: 647–676. 11181971 10.1146/annurev.physiol.63.1.647

[3] King DP, Zhao Y, Sangoram AM, Wilsbacher LD, Tanaka M, Antoch MP, et al Positional cloning of the mouse circadian clock gene. Cell. 1997; 89: 641–653. 9160755 10.1016/s0092-8674(00)80245-7 PMC3815553

[4] Gekakis N, Staknis D, Nguyen HB, Davis FC, Wilsbacher LD, King DP, et al Role of the CLOCK protein in the mammalian circadian mechanism. Science. 1998; 280: 1564–1569. 10.1126/science.280.5369.1564 9616112

[5] Bunger MK, Wilsbacher LD, Moran SM, Clendenin C, Radcliffe LA, Hogenesch JB, et al Mop3 is an essential component of the master circadian pacemaker in mammals. Cell. 2000; 103: 1009–1017. 11163178 10.1016/s0092-8674(00)00205-1 PMC3779439

[6] Kume K, Zylka MJ, Sriram S, Shearman LP, Weaver DR, Jin X, et al mCRY1 and mCRY2 Are Essential Components of the Negative Limbof the Circadian Clock Feedback Loop. Cell. 1999; 98: 193–205. 10428031 10.1016/s0092-8674(00)81014-4

[7] Harada Y, Sakai M, Kurabayashi N, Hirota T, Fukada Y. Ser-557-phosphorylated mCRY2 is degraded upon synergistic phosphorylation by glycogen synthase kinase-3 beta. J Biol Chem. 2005; 280: 31714–31721. 10.1074/jbc.M506225200 15980066

[8] Lamia KA, Sachdeva UM, DiTacchio L, Williams EC, Alvarez JG, Egan DF, et al AMPK Regulates the Circadian Clock by Cryptochrome Phosphorylation and Degradation. Science. 2009; 326: 437–440. 10.1126/science.1172156 19833968 PMC2819106

[9] Gao P, Yoo S-H, Lee K-J, Rosensweig C, Takahashi JS, Chen BP, et al Phosphorylation of the Cryptochrome 1 C-terminal Tail Regulates Circadian Period Length. J Biol Chem. 2013; 288: 35277–35286. 10.1074/jbc.M113.509604 24158435 PMC3853276

[10] Kurabayashi N, Hirota T, Sakai M, Sanada K, Fukada Y. DYRK1A and Glycogen Synthase Kinase 3, a Dual-Kinase Mechanism Directing Proteasomal Degradation of CRY2 for Circadian Timekeeping. Mol Cell Biol. 2010; 30: 1757–1768. 10.1128/MCB.01047-09 20123978 PMC2838083

[11] Stojkovic K, Wing SS, Cermakian N. A central role for ubiquitination within a circadian clock protein modification code. Front Mol Neurosci. 2014; 7: 69 10.3389/fnmol.2014.00069 25147498 PMC4124793

[12] Siepka SM, Yoo S-H, Park J, Song W, Kumar V, Hu Y, et al Circadian mutant Overtime reveals F-box protein FBXL3 regulation of cryptochrome and period gene expression. Cell. 2007; 129: 1011–1023. 10.1016/j.cell.2007.04.030 17462724 PMC3762874

[13] Busino L, Bassermann F, Maiolica A, Lee C, Nolan PM, Godinho SI, et al SCFFbxl3 controls the oscillation of the circadian clock by directing the degradation of cryptochrome proteins. Science. 2007; 316: 900–904. 10.1126/science.1141194 17463251

[14] Godinho SIH, Maywood ES, Shaw L, Tucci V, Barnard AR, Busino L, et al The after-hours mutant reveals a role for Fbxl3 in determining mammalian circadian period. Science. 2007; 316: 897–900. 10.1126/science.1141138 17463252

[15] Hirano A, Yumimoto K, Tsunematsu R, Matsumoto M, Oyama M, Kozuka-Hata H, et al FBXL21 Regulates Oscillation of the Circadian Clock through Ubiquitination and Stabilization of Cryptochromes. Cell. 2013; 152: 1106–1118. 10.1016/j.cell.2013.01.054 23452856

[16] Shi G, Xing L, Liu Z, Qu Z, Wu X, Dong Z, et al Dual roles of FBXL3 in the mammalian circadian feedback loops are important for period determination and robustness of the clock. Proc Natl Acad Sci U S A. 2013; 110: 4750–4755. 10.1073/pnas.1302560110/-/DCSupplemental/pnas.201302560SI.pdf 23471982 PMC3606995

[17] Yoo S-H, Mohawk JA, Siepka SM, Shan Y, Huh SK, Hong H-K, et al Competing E3 ubiquitin ligases govern circadian periodicity by degradation of CRY in nucleus and cytoplasm. Cell. 2013; 152: 1091–1105. 10.1016/j.cell.2013.01.055 23452855 PMC3694781

[18] Nicholson B, Suresh Kumar KG. The multifaceted roles of USP7: new therapeutic opportunities. Cell Biochem Biophys. 2011; 60: 61–68. 10.1007/s12013-011-9185-5 21468693

[19] Papp SJ, Huber AL, Jordan SD, Kriebs A, Nguyen M, Moresco JJ. DNA damage shifts circadian clock time via Hausp-dependent Cry1 stabilization. eLife. 2015; 10.7554/eLife.04883.001 PMC4352707 25756610

[20] Baralle M, Buratti E, Baralle FE. The role of TDP-43 in the pathogenesis of ALS and FTLD. Biochem Soc Trans. 2013; 41: 1536–1540. 10.1042/BST20130186 24256250

[21] Verma A, Tandan R. RNA quality control and protein aggregates in amyotrophic lateral sclerosis: A review. Muscle Nerve. 2013; 47: 330–338. 10.1002/mus.23673 23381726

[22] Lee C, Etchegaray JP, Cagampang FR, Loudon AS, Reppert SM. Posttranslational mechanisms regulate the mammalian circadian clock. Cell. 2001; 107: 855–867. 11779462 10.1016/s0092-8674(01)00610-9

[23] Lamia KA, Papp SJ, Yu RT, Barish GD, Uhlenhaut NH, Jonker JW, et al Cryptochromes mediate rhythmic repression of the glucocorticoid receptor. Nature. 2012; 480: 552–556. 10.1038/nature10700 PMC3245818 22170608

[24] Tong X, Zhang D, Guha A, Arthurs B, Cazares V, Gupta N, et al PLOS ONE: CUL4-DDB1-CDT2 E3 Ligase Regulates the Molecular Clock Activity by Promoting Ubiquitination-Dependent Degradation of the Mammalian CRY1. PLoS ONE. 2015; 10: e0139725 10.1371/journal.pone.0139725.s004 26431207 PMC4592254

[25] Lee M-H, Lozano G. Regulation of the p53-MDM2 pathway by 14-3-3 σ and other proteins. Semin Cancer Biol. 2016; 16: 225–234. 10.1016/j.semcancer.2006.03.009 16697215

[26] Tong X, Buelow K, Guha A, Rausch R, Yin L. USP2a protein deubiquitinates and stabilizes the circadian protein CRY1 in response to inflammatory signals. J Biol Chem. 2012; 287: 25280–25291. 10.1074/jbc.M112.340786 22669941 PMC3408137

[27] Hu M, Li P, Li M, Li W, Yao T, Wu J-W, et al Crystal structure of a UBP-family deubiquitinating enzyme in isolation and in complex with ubiquitin aldehyde. Cell. 2002; 111: 1041–1054. 12507430 10.1016/s0092-8674(02)01199-6

[28] Colland F, Formstecher E, Jacq X, Reverdy C, Planquette C, Conrath S, et al Small-molecule inhibitor of USP7/HAUSP ubiquitin protease stabilizes and activates p53 in cells. Mol Cancer Ther. 2009; 8: 2286–2295. 10.1158/1535-7163.MCT-09-0097 19671755

[29] Ventii KH, Wilkinson KD. Protein partners of deubiquitinating enzymes. Biochem J. 2008; 414: 161 10.1042/BJ20080798 18687060 PMC2724835

[30] Fang L, Yang Z, Zhou J, Tung J-Y, Hsiao C-D, Wang L, et al Circadian clock gene CRY2 degradation is involved in chemoresistance of colorectal cancer. Mol Cancer Ther. 2015; 10.1158/1535-7163.MCT-15-0030 PMC4458447 25855785

[31] Hirota T, Lee JW, St John PC, Sawa M, Iwaisako K, Noguchi T, et al Identification of Small Molecule Activators of Cryptochrome. Science. 2012; 337: 1094–1097. 10.1126/science.1223710 22798407 PMC3589997

[32] Hirano A, Kurabayashi N, Nakagawa T, Shioi G, Todo T, Fukada Y. In Vivo Role of Phosphorylation of Cryptochrome 2 in the Mouse Circadian Clock. Mol Cell Biol. 2014; 34: 4464–4473. 10.1128/MCB.00711-14 25288642 PMC4248739

[33] Hanson KA, Kim SH, Tibbetts RS. RNA-binding proteins in neurodegenerative disease: TDP-43 and beyond. WIREs RNA. 2011; 3: 265–285. 10.1002/wrna.111 22028183 PMC3766724

[34] Gallego M, Virshup DM. Post-translational modifications regulate the ticking of the circadian clock. Nat Rev Mol Cell Biol. 2007; 8: 139–148. 10.1038/nrm2106 17245414

[35] Komander D, Rape M. The Ubiquitin Code. Annu Rev Biochem. 2012; 81: 203–229. 10.1146/annurev-biochem-060310-170328 22524316

[36] Yang Y, Duguay D, Bedard N, Rachalski A, Baquiran G, Na CH, et al Regulation of behavioral circadian rhythms and clock protein PER1 by the deubiquitinating enzyme USP2. Biology Open. 2012; 1: 789–801. 10.1242/bio.20121990 23213472 PMC3507220

[37] Scoma HD, Humby M, Yadav G, Zhang Q, Fogerty J, Besharse JC, et al The De-Ubiquitinylating Enzyme, USP2, Is Associated with the Circadian Clockwork and Regulates Its Sensitivity to Light. PLoS ONE. 2011; 6: e25382 10.1371/journal.pone.0025382 21966515 PMC3179520

[38] Kim J, D'Annibale S, Magliozzi R, Low TY, Jansen P, Shaltiel IA, et al USP17- and SCF TrCP-Regulated Degradation of DEC1 Controls the DNA Damage Response. Mol Cell Biol. 2014; 34: 4177–4185. 10.1128/MCB.00530-14 25202122 PMC4248718

[39] Sato Y, Goto E, Shibata Y, Kubota Y, Yamagata A, Goto-Ito S, et al Structures of CYLD USP with Met1- or Lys63-linked diubiquitin reveal mechanisms for dual specificity. Nat Struct Mol Biol. 2015; 22: 222–229. 10.1038/nsmb.2970 25686088

[40] Liscic RM, Grinberg LT, Zidar J, Gitcho MA, Cairns NJ. ALS and FTLD: two faces of TDP-43 proteinopathy. Eur J Neurol. 2008; 15: 772–780. 10.1111/j.1468-1331.2008.02195.x 18684309 PMC2801606

[41] Barthlen GM, Lange DJ. Unexpectedly severe sleep and respiratory pathology in patients with amyotrophic lateral sclerosis. Eur J Neurol. 2000; 7: 299–302. 10886313 10.1046/j.1468-1331.2000.00044.x

[42] Soekadar SR, Born J, Birbaumer N, Bensch M, Halder S, Murguialday AR, et al Fragmentation of slow wave sleep after onset of complete locked-in state. J Clin Sleep Med. 2013; 9: 951–953. 10.5664/jcsm.3002 23997708 PMC3746723

[43] Heintzen C, Liu Y. The Neurospora crassa Circadian Clock. Advances in Genetics. 2007; 58: 25–66. 10.1016/S0065-2660(06)58002-2 17452245

[44] Hurley JM, Larrondo LF, Loros JJ, Dunlap JC. Conserved RNA Helicase FRH Acts Nonenzymatically to Support the Intrinsically Disordered Neurospora Clock Protein FRQ. Mol Cell. 2013; 52: 832–843. 10.1016/j.molcel.2013.11.005 24316221 PMC3900029

[45] St John PC, Hirota T, Kay SA, Doyle FJ. Spatiotemporal separation of PER and CRY posttranslational regulation in the mammalian circadian clock. Proc Natl Acad Sci U S A. 2014; 111: 2040–2045. 10.1073/pnas.1323618111 24449901 PMC3918757

[46] Kon N, Hirota T, Kawamoto T, Kato Y, Tsubota T, Fukada Y, et al Activation of TGF-β/activin signalling resets the circadian clock through rapid induction of Dec1 transcripts. Nat Cell Biol. 2008; 10: 1463–1469. 10.1038/ncb1806 19029909

