# Supplementary material for: USP7 and TDP-43: Pleiotropic Regulation of Cryptochrome Protein Stability Paces the Oscillation of the Mammalian Circadian Clock
Source: PLoS One. 2016 Apr 28;11(4):e0154263. doi: 10.1371/journal.pone.0154263 (PMC4849774; doi:10.1371/journal.pone.0154263)
Supplement: S1 Table — Proteins with Mascot score above 100 in CRY1 or CRY2 interactome analysis were listed. (DOCX) [file pone.0154263.s009.docx]

**S1 Table Proteins co-purified with either CRY1 or CRY2.**

Proteins with Mascot scores above 100 in CRY1 or CRY2 interactome analysis were listed.

**S1 Table CRY interacting proteins**

| Proteins | MS Score | |  | Coverage (%) | |
| --- | --- | --- | --- | --- | --- |
|  | CRY1 | CRY2 |  | CRY1 | CRY2 |
| GCN1 general control of amino-acid synthesis 1-like 1 | 687 | 536 |  | 10.295769 | 5.1666042 |
| ras GTPase-activating-like protein IQGAP1 | 485 | 1116 |  | 14.302957 | 11.466505 |
| large proline-rich protein BAG6 isoform 1 | 448 | 1100 |  | 10.138648 | 7.8856152 |
| polymerase I and transcript release factor | 336 | 852 |  | 18.877551 | 20.918367 |
| E3 ubiquitin-protein ligase NEDD4 | 308 | 74 |  | 10.71025 | 3.4949267 |
| protein phosphatase 1A | 285 | 258 |  | 15.706806 | 15.706806 |
| DNA topoisomerase 2-alpha | 248 | 109 |  | 2.8795811 | 2.1596858 |
| BTB/POZ domain-containing protein KCTD17 | 244 | 109 |  | 19.594594 | 5.0675675 |
| DNA damage-binding protein 1 | 215 | 321 |  | 6.2280701 | 5.7894736 |
| insulin-like growth factor 2 mRNA-binding protein 3 | 204 | 103 |  | 10.708117 | 3.9723661 |
| BTB/POZ domain-containing protein KCTD5 | 200 | 56 |  | 20.940170 | 6.8376068 |
| TAR DNA-binding protein 43 isoform 1 | 200 | 375 |  | 10.144927 | 9.4202898 |
| transcription intermediary factor 1-beta | 188 | 96 |  | 12.230215 | 5.875299 |
| double-stranded RNA-binding protein Staufen homolog 1 isoform 2 | 160 | 122 |  | 11.247443 | 5.1124744 |
| elongator complex protein 1 | 154 | 51 |  | 5.8514628 | 0.8252063 |
| S-phase kinase-associated protein 1 | 151 | 327 |  | 55.214723 | 7.361963 |
| stress-induced-phosphoprotein 1 | 131 | 126 |  | 9.3922651 | 6.0773480 |
| nucleolar RNA helicase 2 | 125 | 36 |  | 4.1128084 | 3.1727379 |
| la-related protein 1 | 118 | 52 |  | 5.5037313 | 1.5858208 |
| ATP-dependent RNA helicase DDX3X | 115 | 371 |  | 8.6102719 | 14.048338 |
| insulin-like growth factor 2 mRNA-binding protein 1 | 115 | 83 |  | 5.1993067 | 4.6793760 |
| exportin-5 | 113 | 58 |  | 1.4119601 | 0.8305647 |
| heme oxygenase 1 | 107 | 106 |  | 9.3425605 | 12.110726 |
| ubiquitin carboxyl-terminal hydrolase 7 | 107 | 49 |  | 8.7941976 | 1.0879419 |
| exocyst complex component 4 | 84 | 150 |  | 1.4358974 | 1.4358974 |
| importin subunit beta-1 | 81 | 106 |  | 5.022831 | 8.7899543 |
| protein timeless homolog isoform 2 | 70 | 127 |  | 3.0910609 | 5.0125313 |
| synaptic vesicle membrane protein VAT-1 homolog | 55 | 323 |  | 8.6206896 | 7.1428571 |
| RING finger protein 126 | 49 | 117 |  | 5.4313099 | 5.4313099 |
| guanine nucleotide-binding protein subunit beta-2-like 1 | 36 | 137 |  | 7.5709779 | 4.1009463 |
| E3 ubiquitin-protein ligase TRIM32 | 32 | 106 |  | 2.2900763 | 2.2900763 |
| myristoylated alanine-rich C-kinase substrate | 30 | 178 |  | 6.1488673 | 15.857605 |
