# Supplementary material for: USP7 and TDP-43: Pleiotropic Regulation of Cryptochrome Protein Stability Paces the Oscillation of the Mammalian Circadian Clock
Source: PLoS One. 2016 Apr 28;11(4):e0154263. doi: 10.1371/journal.pone.0154263 (PMC4849774; doi:10.1371/journal.pone.0154263)
Supplement: S5 Table — (DOCX) [file pone.0154263.s013.docx]

**S5 Table Detailed transfection conditions for the degradation assay.**

| Figures | plasmids | DNA amounts | Transfection | incubation time |
| --- | --- | --- | --- | --- |
| Fig 3B, S3 Fig | LUC (empty, CRY1 or CRY2) | 100 | Lipofectamine | 48 hr |
|  | pcDNA3 (empty or USP7) | 200 |  |  |
| Fig 3E, S5C Fig | LUC (empty or CRY1) | 5 | PEI | 72 hr |
|  | pSilencer3.1-H1 (scramble or *Usp7* sh) | 400 |  |  |
|  | pcDNA3 (empty) | 595 |  |  |
| Fig 3E | LUC (CRY2) | 100 | Lipofectamine | 48 hr |
|  | pSilencer3.1-H1 (scramble or *Usp7* sh) | 400 |  |  |
| Fig 3F | LUC (CRY2) | 5 | PEI | 72 hr |
|  | pSilencer3.1-H1 (scramble or *Fbxl3* sh) | 500 |  |  |
|  | pcDNA3 (empty or USP7) | 500 |  |  |
|  | pcDNA3 (empty) | 995 |  |  |
|  | LUC (empty, CRY1 or CRY2) | 5 |  |  |
| Fig 5D, S7 Fig | pcDNA3.1 (empty or TDP-43) | 500 | PEI | 48 hr |
|  | pcDNA3 (empty) | 495 |  |  |
|  | pcDNA3.1 (empty) | 1000 |  |  |
|  | CRY2-LUC | 100 |  |  |
| Fig 5E | pSilencer3.1-H1 (scramble or *Tdp43* sh) | 400 | Lipofectamine | 48 hr |
|  | LUC (CRY2) | 5 |  |  |
| Fig 5F | pSilencer3.1-H1 (scramble or *Fbxl3* sh) | 500 | PEI | 72 hr |
|  | pcDNA3.1 (empty TDP-43) | 500 |  |  |
|  | pcDNA3 (empty) | 995 |  |  |
